# Identification of a new member of Mortaparib class of inhibitors that target mortalin and PARP1

**DOI:** 10.3389/fcell.2022.918970

**Published:** 2022-09-12

**Authors:** Hazna Noor Meidinna, Seyad Shefrin, Anissa Nofita Sari, Huayue Zhang, Jaspreet Kaur Dhanjal, Sunil C. Kaul, Durai Sundar, Renu Wadhwa

**Affiliations:** ^1^ AIST-INDIA DAILAB, National Institute of Advanced Industrial Science & Technology (AIST), Tsukuba, Japan; ^2^ Department of Biochemical Engineering and Biotechnology, Indian Institute of Technology (IIT) Delhi, New Delhi, India; ^3^ Department of Computational Biology, Indraprastha Institute of Information Technology Delhi, New Delhi, India

**Keywords:** mortalin, PARP1, mortalin-p53 interaction, triazole derivative, anticancer drug

## Abstract

Mortalin, a heat shock family protein enriched in cancer cells, is known to inactivate tumor suppressor protein p53. Abrogation of mortalin-p53 interaction and reactivation of p53 has been shown to trigger growth arrest/apoptosis in cancer cells and hence, suggested to be useful in cancer therapy. In this premise, we earlier screened a chemical library to identify potential disruptors of mortalin-p53 interaction, and reported two novel synthetic small molecules (5-[1-(4-methoxyphenyl) (1,2,3,4-tetraazol-5-yl)]-4-phenylpyrimidine-2-ylamine) and (4-[(1E)-2-(2-phenylindol-3-yl)-1-azavinyl]-1,2,4-triazole) called Mortaparib and Mortaparib^Plus^, respectively. These compounds were shown to possess anticancer activity that was mediated through targeting mortalin and PARP1 proteins, essential for cancer cell survival and proliferation. Here, we report characterization of the third compound, {4-[(4-amino-5-thiophen-2-yl-1,2,4-triazol-3-yl)sulfanylmethyl]-N-(4-methoxyphenyl)-1,3-thiazol-2-amine}, isolated in the same screening. Extensive computational and molecular analyses suggested that the new compound has the capability to interact with mortalin, p53, and PARP1. We provide evidence that this new compound, although required in high concentration as compared to the earlier two compounds (Mortaparib and Mortaparib^Plus^) and hence called Mortaparib^Mild^, also downregulates mortalin and PARP1 expression and functions in multiple ways impeding cancer cell proliferation and migration characteristics. Mortaparib^Mild^ is a novel candidate anticancer compound that warrants further experimental and clinical attention.

## Introduction

Cancer is an extremely complex disease showing exponential increase in global incidence and hence continues to demand new diagnostic, preventive, and treatment modalities. Search for new drug targets and drugs is defined as a priority field in oncology and has led to several FDA-approved drugs that are extensively used in chemotherapy. However, high cost of these drugs, their adverse effects, and drug resistance leading to tumor relapse have been the major concerns leading to continuous research and drug development at various levels. Mortalin, a member of the Hsp70 family of proteins, enriched in many types of cancers, has been shown to be involved in multiple ways in the process of carcinogenesis (Dundas et al., 2005; Wadhwa et al., 2006; Yi et al., 2008; Yun et al., 2017; Xu et al., 2020; Meng et al., 2021; Teng et al., 2021; Wei et al., 2021; Wu et al., 2021; Yang et al., 2021; Zhang et al., 2021). It has been shown to have differential cellular distribution in normal and cancer cells (Wadhwa et al., 1993). Whereas normal cells exhibit pan-cytoplasmic distribution of mortalin, most cancer cells show its perinuclear distribution. Furthermore, induction of senescence in cancer cells was shown to involve shift in mortalin distribution from perinuclear to pan-cytoplasmic localization (Fujii et al., 1995; Wadhwa et al., 2004; Widodo et al., 2007a; Garg et al., 2020).

Mortalin, with its essential mitochondrial and extra-mitochondrial functions, plays multiple roles in proliferation, migration and stress response of cells (Craig et al., 1989; Wadhwa et al., 1993; Merrick et al., 1997; Wadhwa et al., 2002b; Wadhwa et al., 2002c; Kaul et al., 2002; Kaul et al., 2007; Deocaris et al., 2009; Londono et al., 2012; Mylonis et al., 2017; Wu et al., 2020; Pagliarone et al., 2021). Overexpression of mortalin has been shown to accelerate carcinogenesis by stimulating cell proliferation, epithelial-mesenchymal transition (EMT) program, and cancer cell stemness (Yun et al., 2017; Xu et al., 2020; Wei et al., 2021). Besides its essential role in maintaining the mitochondrial integrity (Czarnecka et al., 2006; Havalova et al., 2021), mortalin has been shown to interact with several proteins and modulate their functions in control of cell division and migration (Wadhwa et al., 1998; Mizukoshi et al., 1999; Sacht et al., 1999; Takano et al., 2001; Schwarzer et al., 2002; Wadhwa et al., 2003; Chen et al., 2014). It has been demonstrated to interact with the key tumor suppressor protein p53, functionally inactivated in a large majority of cancers (Wadhwa et al., 1998; Benbrook et al., 2014; Sane et al., 2014; Pham et al., 2019; Benbrook, 2022). The interaction of mortalin and p53 in the cytoplasm results in cytoplasmic retention and hence the inactivation of transcriptional activation function of p53 in cancer cells (Wadhwa et al., 1998; Kaul et al., 2001; Wadhwa et al., 2002c; Walker et al., 2006). On the other hand, mortalin-p53 interaction in the nucleus has been shown to control centrosome duplication (Ma et al., 2006) and activate telomerase (Ryu et al., 2014). Mortalin compromised cancer cells have been shown to undergo growth arrest and apoptosis (Wadhwa et al., 2004; Wadhwa et al., 2016).

We had earlier demonstrated that the knockdown of mortalin in human immortalized cells that lacked functional p53 and pRB, and possessed activated telomerase induced permanent growth arrest (Wadhwa et al., 2004). Mortalin-targeting adeno-oncolytic virus was shown to be selectively cytotoxic to human cancer cells *in vitro* and induced apoptosis in *in vivo* tumor models (Yoo et al., 2010). Mortalin overexpression, on the other hand, enhanced the malignant properties of cancer cells in breast xenograft models (Yi et al., 2008; Yoo et al., 2010). Clinical relevance of mortalin overexpression in hepatocellular carcinoma (HCC) was supported by the study in which advanced aggressive stages of HCC and early recurrence were correlated with a higher level of mortalin expression. Mortalin was shown to promote EMT and angiogenesis (Chen et al., 2014). Most cancer cells show dysregulation of apoptotic function that may involve inactivation of innate tumor suppression, apoptosis-inducing proteins and activation of oncogenes including mutant p53 (Kato et al., 2003; Lowe et al., 2004). Lu et al. (2011) and Lu et al. (2011) validated significance of mortalin overexpression in HCC using mutant p53 harboring HCC cell lines. shRNA-mediated mortalin silencing in these cells could induce tumor cell-specific apoptosis. Hu et al. (2018) supported the involvement of mortalin in hepatitis, cirrhosis and hepatocellular carcinoma. Yang et al. (2013) validated its role in progression of ovarian cancers by using lentivirus driven overexpression and its knockdown. By a variety of assays, it was shown to promote the G1 transition. Mortalin-compromised cells showed increase in C-myc and Cyclin-D1, and decrease in Cyclin-B1, p-c-Raf and p-ERK1/2. Mortalin-overexpressing cells showed the opposite effects. Furthermore, mortalin overexpression was shown to contribute to drug resistance in ovarian cancer cells (Yang et al., 2013). Dai et al. (2021) showed that mortalin is highly expressed in cisplatin-resistant gastro-intestinal cancer cells. Knockdown of mortalin resulted in metabolic reprogramming of these cells and re-sensitized them to cisplatin *in vitro* and *in vivo*. These reports have suggested that the targeting of mortalin-p53 interaction and reactivation of p53 functions by natural and synthetic compounds is a viable cancer therapeutic strategy.

Poly (ADP-Ribose) Polymerase 1 (PARP1) is the first characterized member of the PARP family that plays role in DNA damage response and in the maintenance of genomic integrity (Pascal, 2018). It is a 116-kDa nuclear protein that senses DNA strand breaks and recruits DNA repair machinery involving its poly (ADP)ribosylation (PARylation) activity. PARP1 catalyses the polymerization of ADP-ribose units resulting in the attachment of PAR polymers to itself or other target proteins to facilitate DNA damage repair (Wang et al., 2019). Thus, PARP1 has been considered as a pharmacological target for the treatment of cancers that have DNA repair defects (Ray Chaudhuri and Nussenzweig, 2017; Sari et al., 2020).

Several natural and synthetic compounds have earlier been shown to disrupt mortalin-p53 interaction. Most of these compounds could relocate and reactivate the tumor suppressor function of p53, resulting in apoptosis or growth arrest in cancer cells (Wadhwa et al., 2000; Wadhwa et al., 2002a; Wadhwa et al., 2006; Widodo et al., 2007b; Lu W. J. et al., 2011; Grover et al., 2012; Nigam et al., 2015; Wadhwa et al., 2016; Bhargava et al., 2018; Radhakrishnan et al., 2021). Based on these reports, mortalin-p53 targeting was considered as a viable drug discovery assay. A library of 12,000 compounds was screened by double visual checkpoint, i.e., 1) shift in mortalin distribution from perinuclear to pan-cytoplasmic and 2) nuclear translocation and reactivation of wild type p53 function. We recently reported two novel small molecules, named Mortaparib (Putri et al., 2019) and Mortaparib^Plus^ (Elwakeel et al., 2021; Sari et al., 2021), that inhibited mortalin-p53 interaction and caused reactivation of p53 function. In addition, they also caused inactivation of PARP1 by binding to its catalytic site wherein Olaparib (an established anti-PARP1 drug has been crystallized) binds resulting in the growth arrest/apoptosis in cancer cells. Mortaparib^Plus^ was also shown to activate p73-and CARF-mediated growth arrest (Elwakeel et al., 2021; Sari et al., 2021). Herein, we report a third small molecule (Mortaparib^Mild^) as a potential abrogator of mortalin-p53 interaction, with computational and experimental evidence to its anticancer activity.

## Materials and methods

### Molecular docking of Mortaparib^Mild^ with p53, mortalin, and PARP1

**FIGURE 1:**
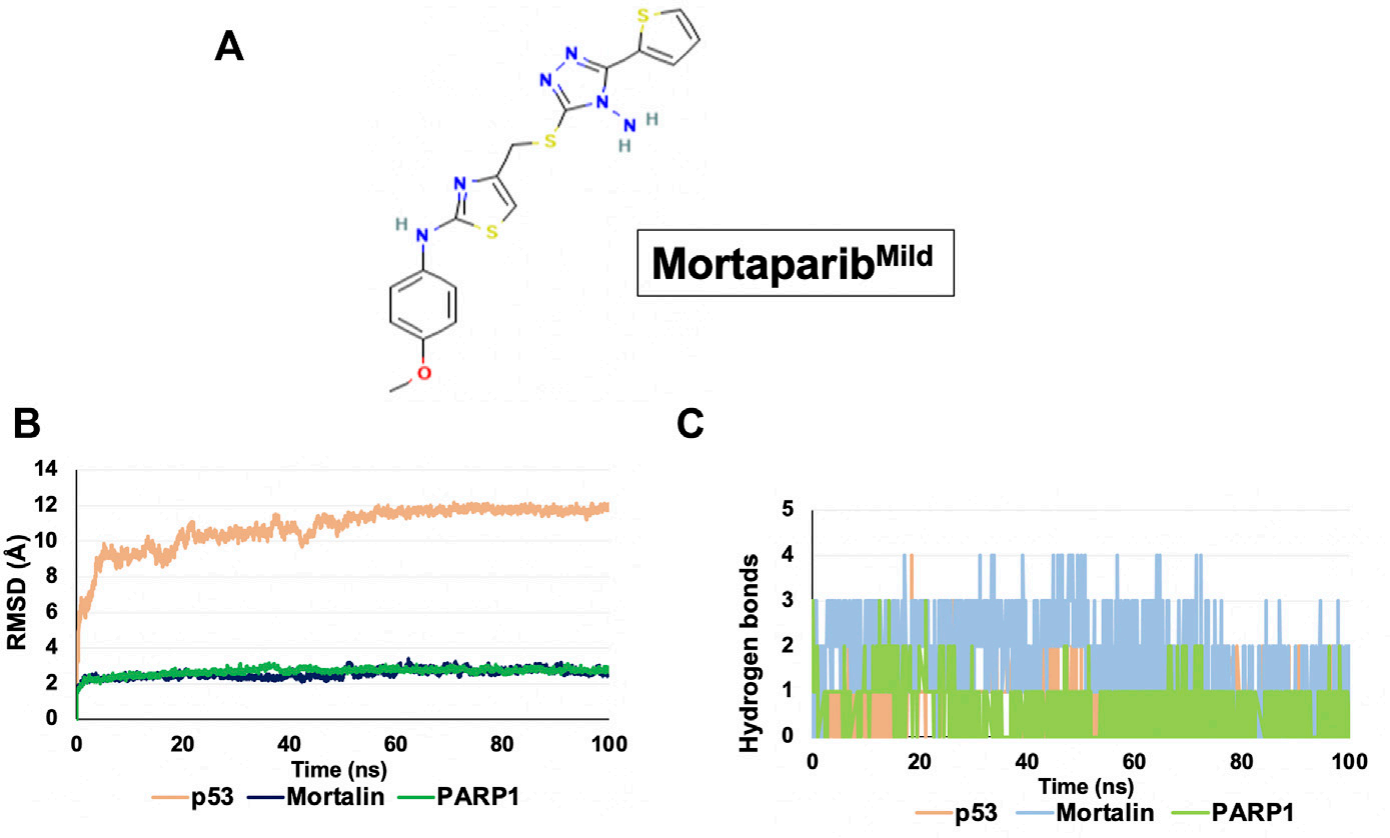
Structure and binding dynamics of Mortaparib^Mild^ with target proteins. **(A)** Molecular structure of Mortaparib^Mild^. **(B)** RMSD plot of Mortaparib^Mild^ interaction with p53, mortalin, and PARP1. **(C)** Hydrogen bond plot of Mortaparib^Mild^ interaction with p53, mortalin, and PARP1.

The PDB structures were obtained for the target proteins - p53 (PDB ID: 1OLG), mortalin (PDB ID: 4KBO), and PARP1 (PDB ID: 4ZZZ) (Clore et al., 1994; Amick et al., 2014; Papeo et al., 2015). The 3D structure of Mortaparib^Mild^ was downloaded from the PubChem repository (CID: 91464252) (Figure 1A). The protein structures were preprocessed using the Protein Preparation module, and the ligand structure was prepared using the Ligprep module in the Schrodinger Maestro suite. Docking grids were generated around the mortalin binding region of p53 (312–352), the p53 binding region of mortalin (253–282), and the Olaparib-binding site in the catalytic domain of PARP1 (862–880) (Kaul et al., 2001; Papeo et al., 2015). Flexible docking was done using the Extra-precision algorithm of the Glide docking program (Friesner et al., 2006).

### Molecular dynamics simulations

Desmond module of Schrodinger software was used for performing the simulations (Schrödinger 2020). The docked protein-ligand complexes were solvated using the “system builder” program of Desmond using a predefined TIP3P water model. In the boundary conditions option, an orthorhombic periodic boundary was set up to give the shape and size of the box buffered at a distance of 10 Å, and then ions (Na+/Cl−) was added to every system depending on the complex for balancing the charge. After building the solvated protein-ligand complex systems, the energy of the prepared systems was minimized through small steps Brownian dynamics for 20 ps at low-temperature (10 K) in NVT ensemble to remove steric clashes (Harder et al., 2016). Further, the minimized systems were equilibrated in seven steps in NVT and NPT ensembles using the relaxation protocol defined in the Desmond Schrodinger suite. Finally, the production simulations were performed for 100 ns in the NPT ensemble with a time step of 2 fs. The pressure and temperature of the systems were kept at 1 atmospheric pressure (using Martyna–Tobias–Kelin barostat) with a relaxation time of 2 ps and 300 K temperature (using Nose–Hoover chain thermostat) with a relaxation time of 1 ps, respectively. The cutoff radius for short-range Coulombic interactions was set to 9 Å. No restraints were added to any molecule and all other options were set to default.

### Analysis of the simulation trajectories

The MD trajectories were analyzed using the “Simulation Event Analysis” module in the Schrodinger suite (Bowers et al., 2006). Root Mean Square Deviation (RMSD) of protein–Mortaparib^Mild^ structures were analyzed as a function of time to investigate the stability of the protein-ligand complexes. The number of hydrogen bonds between the ligand and proteins was calculated throughout the simulation time. The RMSD of ligands was also calculated and compared to investigate their flexibility, binding inside the active pocket of protein, and their stability throughout the simulation (Liu and Kokubo, 2017). The average structure was finally visualized using the PyMOL suite of the Schrodinger package.

### Calculation of binding free energy

The average representative structure was chosen from the simulation trajectories using the “Average Structure” module of the Schrodinger suite. Prime MM/GBSA was used for binding free energy calculation (Li et al., 2011).

The equation used for the calculation was:

$$\text{MM/GBSA }\Delta {\text{G}}_{\text{bind}}\mathrm{=\Delta}{\text{G}}_{\text{complex}}-\left(\Delta {\text{G}}_{\text{receptor}}\mathrm{+\Delta}{\text{G}}_{\text{ligand}}\right)$$

where, ∆G _complex_, ∆G _receptor,_ and ∆G _ligand_ represent the free energies of the complex, receptor, and ligand, respectively. MM/GBSA refers to the binding affinity of the ligand towards the target protein; a more negative value represents stronger affinity (Genheden and Ryde, 2015). The calculated binding free energy values are not absolute due to the inherent limitations of end-point free energy calculation methods.

### Cell culture and reagents

Human cancer cells, colorectal (HCT116 and DLD1), breast (T47D, MCF7, MDA-MB231, and MDA-MB453), cervical (HeLa, ME-180, SKG-II, SKG-IIIb, and CASKI), hepatic (HuH7), lung (A549), bone (U2OS), and the normal fibroblasts (TIG3 and MRC5) (JCRB, Tokyo, Japan) were cultured in Dulbecco’s Modified Eagle’s Medium (DMEM) low glucose with L-glutamine and phenol red (Fujifilm WAKO Pure Chemical Corporation, Osaka, Japan) supplemented with 5% fetal bovine serum (Thermo Fisher Scientific, Japan), 1% penicillin-streptomycin (Invitrogen, Carlsbad, CA, United States) at 37°C in an atmosphere of 5% CO_2_.

### Drug preparation and treatment

Mortaparib^Mild^ (triazole derivative 4-[(4-amino-5-thiophen-2-yl-1,2,4-triazol-3-yl)sulfanylmethyl]-N-(4-methoxyphenyl)-1,3-thiazol-2-amine) (NAMIKI SHOJI Co., Ltd.; Shinjuku, Japan) was dissolved in dimethyl sulfoxide (DMSO) (WAKO, Osaka, Japan) to prepare 50 mM stock. The stock was diluted in a complete cell culture media to obtain working concentrations (1–80 μM). The cells were treated with Mortaparib^Mild^ at 60–70% of confluency for 24–48 h.

### Library screening

Screening was performed from the library consisting of 12,000 synthetic and natural compounds to obtain candidates abrogating mortalin-p53 interactions, as previously described (Putri et al., 2019).

### Short- and long-term cytotoxicity assays

The short- and long-term cytotoxicity of Mortaparib^Mild^ was determined by MTT-based cell viability and colony formation assays in 96-well and 6-well plates, respectively, as described earlier (Putri et al., 2019; Sari et al., 2021). Morphology of control and treated cells was also captured under phase contrast light microscope (Nikon TS100-F, Tokyo, Japan).

### Luciferase reporter assay

Wild type p53 dependent luciferase reporter assay was performed in HCT116 and T47D cells with/without the treatment with Mortaparib^Mild^. Cells (2 × 10^5^/well of 6-well plates) were transfected with PG13-luc (the wild type p53 responsive luciferase-reporter plasmid) using X-tremeGENE HP DNA transfection reagent (Roche, Basel, Switzerland) as described earlier (Sari et al., 2021). Cells, treated with either DMSO or Mortaparib^Mild^ for 24 h, were collected by trypsinization, lysed using the passive lysis buffer (PLB) (Promega, WI, United States), and subjected to luciferase activity assay using the Luciferase Reporter Assay System (Promega, Madison, WI, United States) as described previously (Sari et al., 2021).

### Apoptosis assay

HCT116 cells were seeded in the 6-well plates (2 × 10^5^ cells/well). After 24 h, control and Mortaparib^Mild^-treated cells were harvested along with the floating cells by centrifugation at 3,000 rpm at 4°C for 5 min. The cell pellet was resuspended in 100 µl fresh media and stained with Guava Nexin Reagent (EMD Millipore Corporation, Berlington, MA, United States). Apoptotic cells were quantified and analyzed by Guava PCA-96 System (Luminex Corporation, Austin TX, United States) and FlowJo software (Version 7.6, Flow Jo, LLC, Ashland, OR, United States).

### Cell cycle analysis

Cells were seeded in 6-well plates (2 × 10^5^ cells/well). After 24 h, control and Mortaparib^Mild^-treated cells were harvested, cold (4°C)-centrifuged at 2,000 rpm for 5 min, washed with cold PBS, fixed with 70% ethanol on slow vortex, and kept at −20°C for up to 72 h. The fixed cells were cold (4°C)-centrifuged at 3,000 rpm for 10 min followed by two cycles of cold PBS washing. The cells were stained with Guava Cell Cycle Reagent (4500-0220) (Luminex Corporation, Austin, TX, United States) in the dark for 30 min. RNase-A (1 mg/ml at 37°C for 30 min) treatment was performed to eliminate RNA in the samples. Cell cycle progression analysis was done using Guava PCA-96 System (Luminex Corporation, Austin, TX United States). FlowJo software (Version 7.6, Flow Jo, LLC, Ashland, OR, United States) was used to analyze the flow cytometry data.

### Western blot analysis

Control and Mortaparib^Mild^-treated cells were harvested by trypsinization. Cell pellets were incubated with RIPA Lysis Buffer (Thermo Fisher Scientific, Waltham, MA, United States) supplemented with complete protease inhibitor cocktail (Roche Applied Science, Mannheim, Germany) for 30 min with slow vortex at 4°C. Lysates were centrifuged at 15,000 rpm for 15 min. Protein concentrations in lysates was determined by Bicinchoninic acid (BCA) protein assay (Thermo Fisher Scientific, Waltham, MA, United States). The cell lysates containing 10–20 µg protein were separated in 8–12% SDS-polyacrylamide gel electrophoresis (SDS-PAGE), transferred to a polyvinylidene difluoride (PVDF) membrane (Millipore, Billerica, MA, United States) using a wet transfer [Mini-PROTEAN Tetra Cell (BIO-RAD, California, United States)] for 75 min or a semi dry transfer blotter (ATTO Corporation, Tokyo, Japan) for 65 min. Membrane blocking was done using 3% bovine serum albumin at room temperature for ∼1 h. Blocked membranes were probed with the target protein-specific primary antibodies overnight at 4°C. Primary antibodies included anti-mortalin (37-6) raised in our laboratory; p21^WAF11^ (12D1) (Cell Signaling Technology, Danvers, MA, United States); anti-cleaved PARP1, anti-Poly (ADP-Ribose) Polymer [10H] (Abcam); MMP 3/10 (F-10), PARP-1 (F-2), p53 (DO-1) (Santa Cruz Biotechnology, Paso Robles, CA, United States). The blots were then incubated with horseradish peroxidase (HRP)-conjugated secondary antibodies [anti-rabbit IgG and anti-mouse IgG (Santa Cruz Biotechnology, CA, United States)] and developed using the enhanced chemiluminescence system (GE Healthcare, Buckinghamshire, United Kingdom). Direct-blot™ anti-β-actin antibody (BioLegend) was used as an internal control. The protein band images were analyzed by ImageJ (National Institutes of Health, Bethesda, MD, United States) software.

### Immunocytochemistry

Cells (4 × 10^4^/well) were plated on 18-mm glass coverslips placed in 12-well plates and allowed to settle overnight followed by treatment with Mortaparib^Mild^ for 24 h and then fixed in methanol:acetone (1:1) at 4°C for 5 min. Cells were washed with phosphate buffered saline (PBS) and permeabilized using PBST (PBS with 0.1% Triton X-100) for 10 min, followed by blocking with 2% bovine serum albumin in PBST at room temperature for 1 h. Fixed cells were incubated with primary antibodies as described previously in Western blotting section and others including, phospho-Histone H2A.X (Ser139) (20E3) (Cell Signaling Technology, Danvers, MA, United States), PUMAα/β (H-136) (Santa Cruz Biotechnology, Paso Robles, CA, United States), Bax (B-9) (Santa Cruz Biotechnology, Paso Robles, CA, United States) and anti-Cytochrome C [EPR1327] (Abcam). Immunostaining was visualized by secondary antibody conjugated with fluorochromes including either FITC or Alexa-488 or Alexa-594 (Molecular Probes, Eugene, OR, United States). Hoechst 33,342 (Invitrogen, Molecular Probes, Eugene, OR, United States) was used for nuclear staining. The coverslips were mounted on glass slides and examined under Zeiss Axiovert 200 M microscope with AxioVision 4.6 software (Carl Zeiss, Tokyo, Japan). Several images were captured from each of the three independent experiments. Protein levels represented by the fluorescence signals obtained using ImageJ software (National Institutes of Health, Bethesda, MD, United States). In each image, the mean intensity of the targeted protein per cell was calculated from 10 to 15 cells. The average of mean intensity per cell was determined along with the standard deviation.

### Immunoprecipitation

The indirect co-immunoprecipitation was performed to investigate the effect of Mortaparib^Mild^ on mortalin-p53 interaction. Control and Mortaparib^Mild^-treated cells were harvested, PBS-washed, and lysed using NP-40 lysis buffer. The protein concentrations were measured by BCA assay (Thermo Fisher Scientific, Rockford, IL). Cell lysates containing 400 μg total protein were precleared with normal rabbit IgG (2729; Cell Signaling Technology, MA, United States) and Protein A/G PLUS-Agarose beads (sc-2003) (Santa Cruz Biotechnology, United States) at 4°C with slow rotation for 1 h, followed by centrifugation at 2,500 rpm at 4°C for 5 min. The supernatant was collected and incubated with either anti-mortalin polyclonal antibody raised in our lab, anti-p53 polyclonal antibody (FL-393; Santa Cruz Biotech., United States) or control normal rabbit IgG at 4°C overnight with slow rotation. Fifty microliters of Protein A/G PLUS-Agarose beads was added to the mixture and incubated for 4 h. Immunoprecipitants were collected by centrifugation at 2,500 rpm at 4°C for 5 min. Pellets were washed with NP-40 lysis buffer followed by centrifugation at 2,500 rpm at 4°C for 5 min (4 times). Immunoprecipitants were boiled at 99°C in SDS sample buffer for 10 min, resolved on SDS-PAGE and subjected to Western blotting with anti-p53 mouse monoclonal antibody (DO-1) from Santa Cruz Biotech. (United States) or anti-mortalin monoclonal antibody raised in our lab. Furthermore, to segregate the effect of Mortaparib^Mild^ on mortalin-p53 interaction from its effect on transcription, translation, post-translation and molecular signaling networks in living cells, we treated cell lysates (prepared in NP-40 lysis buffer) with Mortaparib^Mild^. Cell lysate containing 700 μg protein was precleared with normal rabbit IgG and Protein A/G PLUS-Agarose beads as described above. The supernatant was divided equally into two parts and incubated with either 0.1% DMSO (control) or 50 μM Mortaparib^Mild^ at room temperature for 3 h with slow rotation, respectively. The control and Mortaparib^Mild^-treated lysates were then incubated with either anti-mortalin or anti-p53 antibodies at 4°C overnight, as described above. Immunocomplexes were separated with Protein A/G PLUS-Agarose beads, resolved by SDS-PAGE and Western blotting analysis, as described above.

### RNA extraction and Real Time quantitative Polymerase Chain Reaction (RT-qPCR)

Total RNA from DMSO (control) and Mortaparib^Mild^-treated cells was collected by RNeasy mini kit (Qiagen, Stanford Valencia, CA, United States) following the manufacturer’s protocol. Equal amounts of RNA (1 µg) from samples were reverse transcribed into cDNA following the protocol from QuantiTect Reverse Transcription kit (Qiagen, Tokyo, Japan). Real Time quantitative Polymerase Chain Reaction (RT-qPCR) was performed using the protocols for SYBR Select Master Mix (Applied Biosystem, Life Technologies, Foster City, CA, United States). The conditions of RT-qPCR using specific primers (Table 1) were 50°C for 2 min, 95°C for 10 min, followed by 40 cycles (denaturation at 95°C for 15 s, annealing at 60°C for 1 min, and extension at 72°C for 15 s). The relative expression level of target genes was normalized against 18S gene as an internal control.

**TABLE 1:** Primer sequences used for Real Time quantitative Polymerase Chain Reaction (RT-qPCR).

Gene (human)	Primer sequence (5′-3′)
Mortalin forward	AGC​TGG​AAT​GGC​CTT​AGT​CAT
Mortalin reverse	CAG​GAG​TTG​GTA​GTA​CCC​AAA​TC
PARP1 forward	TCA​GCC​TCC​TTG​CTA​CAG​AGG
PARP1 reverse	GGT​CGT​TCT​GAG​CCT​TTA​GGG
18S forward	CAG​GGT​TCG​ATT​CCG​TAG​AG
18S reverse	CCT​CCA​GTG​GAT​CCT​CGT​TA

### Trapping assay

Trapping assay was aimed to fractionate PARP-DNA complexes in different stringency hypotonic buffers (A, B, C, and/or D; Table 2), wherein the tight complex is characterized by fractionation of PARP in higher stringency buffer as described earlier (Murai et al., 2012). Mortaparib^Mild^-treated and control cells were collected by trypsinization and centrifugation (2,500 rpm) at 4°C for 3 min. Supernatants were removed, and pellets were mixed with hypotonic buffers (Table 2), vortexed for 10 min followed by centrifugation at 16,000 rpm at 4°C for 10 min. The collected supernatant was labelled as P1 and pellet was re-suspended with Buffer A. This step was serially repeated in the sequence of buffer A-D. Supernatants from each centrifugation step were collected and labelled as A, B, C, and D. Western blotting analysis was perform using anti-PARP1/2 and anti-histone H3 antibodies.

**TABLE 2:** Composition of trapping assay buffers.

Buffer	Ingredients
Hypotonic buffer	100 mM MES-NaOH pH 6.4, 1 mM EDTA, 0.5 mM MgCl_2_, 30% sucrose in Mili-Q H_2_O
Buffer A	50 mM HEPES-NaOH pH 7.5, 100 mM KCl, 2.5 mM MgCl_2_, 0.05% Triton X-100
Buffer B	50 mM HEPES-NaOH pH 7.5, 250 mM KCl, 2.5 mM MgCl_2_, 0.05% Triton X-100
Buffer C	50 mM HEPES-NaOH pH 7.5, 500 mM KCl, 2.5 mM MgCl_2_, 0.1% Triton X-100
Buffer D	Buffer A, 5 mM CaCl_2_, Micrococcal protease inhibitor three-unit (Roche Diagnostic GmbH, Mannheim, Germany)

### Wound healing assay

Cells (2 × 10^5^/well) were plated in 6-well plates and allowed to make monolayers overnight. The monolayer of cells was manually scraped using a p200 pipette tip to create a linear wound. The cells were washed with PBS and cultured in complete culture medium-supplemented with DMSO (control) or non-toxic concentration (5 µM for HCT116 and T47D) of Mortaparib^Mild^. The migration of cancer cells into the gap was imaged over 0–96 h under a microscope (Nikon TS100-F, Tokyo, Japan).

### Statistical analysis

Data obtained from three or more independent experiments were statistically expressed as mean ± standard deviation. Unpaired *t* test (GraphPad Prism, GraphPad Software, San Diego, CA) was performed to determine statistical significance between the control and experimental samples. Values of *p* > 0.05 (^ns^), *p* ≤ 0.05 (*)‬, *p* ≤ 0.01 (**), *p* ‬≤ 0.001 (***) and *p*‬ ≤ 0.0001 (****) were considered non-significant, statistically significant‬, very significant, highly significant, and extremely significant, respectively.

## Results

### Mortaparib^Mild^ is predicted to interact with p53, mortalin, and PARP1

Molecular docking was performed to analyze the interaction of Mortaparib^Mild^ in the mortalin-binding region of p53, p53-binding region of mortalin, and catalytic site of PARP1. Mortaparib^Mild^ was found to be interacting with 1) the mortalin-binding domain (amino acid residue number: 323–337) of p53 with a docking score of −3.348 kcal/mol, 2) within the p53 binding domain (amino acid residue number: 260–280) of mortalin with a docking score of -2.873 kcal/mol, and 3) with the catalytic binding domain of PARP1 with a docking score of −8.202 kcal/mol, where known drug Olaparib (inhibitor of PARP1) binds. The docking score was calculated for determining the strength of ligand binding with the target protein based on the hydrogen bonding, hydrophobic interactions, ionic interactions, and aromatic ring stacking (Bohm, 1998). Docking scores represent empirical values for relative assessment of ligand binding; higher docking score signifies stronger interaction. Furthermore, each of the protein-drug complexes was subjected to molecular dynamics (MD) simulation for 100 ns that determined the stability of ligand-target protein interaction. RMSD of all the protein-drug complexes converged within 40 ns indicating a stable interaction (Figure 1B). The hydrogen bond plot obtained throughout the simulation revealed that Mortaparib^Mild^ stably interacted with p53, mortalin and PARP1 at the docked site, and formed continuous hydrogen bonds throughout the simulation (Figure 1C). After every 10 ns simulation, frames of all three complexes were extracted and the stable binding of the Mortaparib^Mild^ with p53, mortalin, (Supplementary Figures S1A,B), and PARP1 (Supplementary Figure S2) was visualized.

**FIGURE 2:**
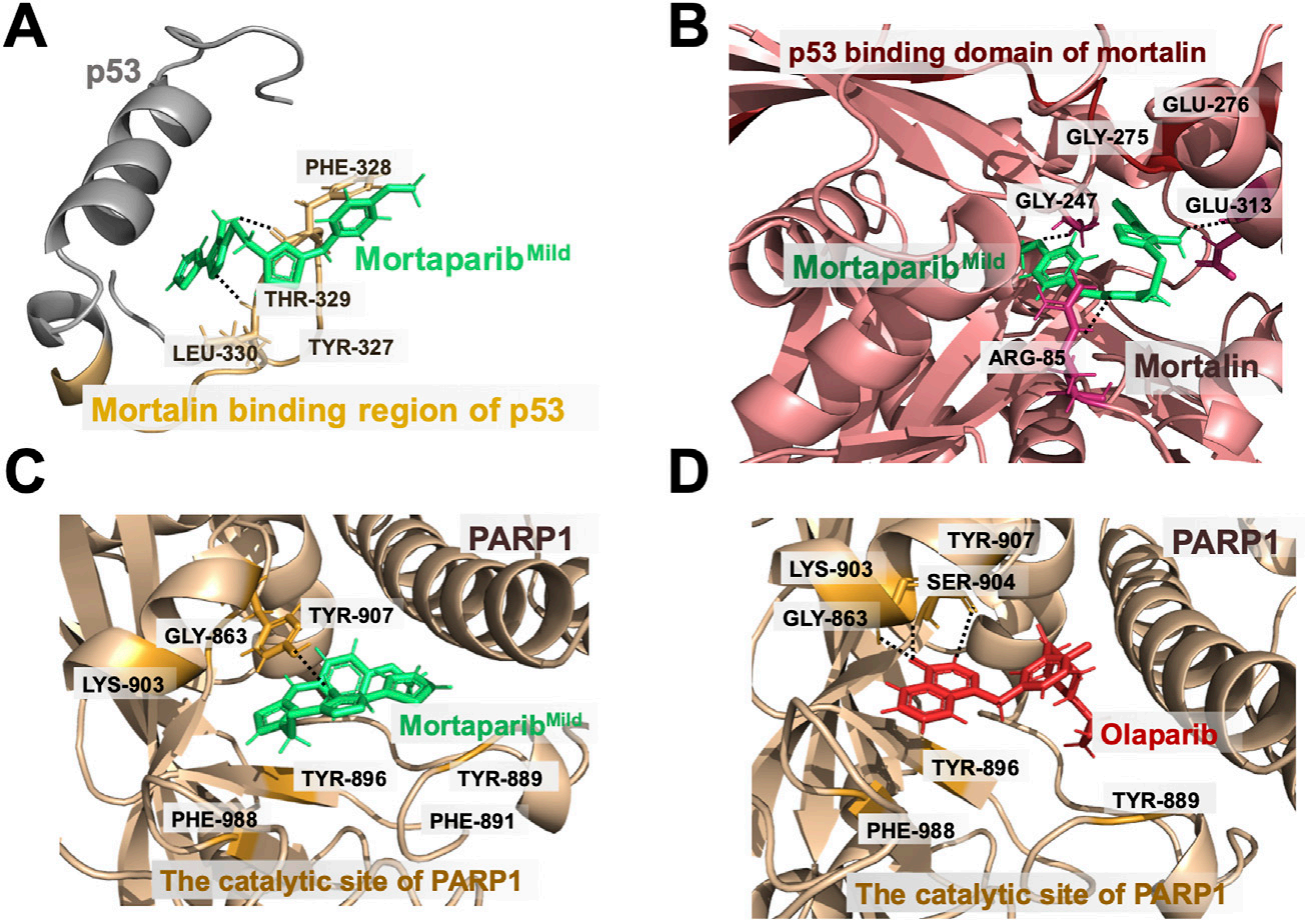
Binding interactions of Mortaparib^Mild^ with target proteins. **(A)** Mortaparib^Mild^ interaction at the mortalin-binding region of p53. **(B)** Mortaparib^Mild^ interaction at the p53-binding region of mortalin. **(C)** Mortaparib^Mild^ interaction with the catalytic domain of PARP1. **(D)** Olaparib interaction with the catalytic domain of PARP1.

Mortaparib^Mild^ formed hydrogen bonds with residues Phe328 and Leu330 and showed hydrophobic interactions with residues Thr329 and Tyr327 in the mortalin binding site of p53 (Figure 2A). All the residues that interacted with Mortaparib^Mild^ were found to be in the mortalin-binding region of p53. From the hydrogen bond occupancy plot of the simulation, it was found that residues Thr329, Phe328, Leu330, and Arg337 were involved for more than 40% of the time during the entire simulation period (Supplementary Figure S3A). Mortaparib^Mild^ interacted with mortalin by forming hydrogen bonds with residues Arg85, Gly247, and Glu313, and had hydrophobic interactions with residues Gly275 and Glu276 in the p53-binding region of mortalin (Figure 2B). Most of the residues were in common with the known p53 binding region involving residues from 253 to 282 in the N-terminal of mortalin (Kaul et al., 2001; Iosefson and Azem, 2010). Moreover, from the hydrogen bond occupancy plot of the simulation, it was found that residues Arg85, Gly247, Glu276, and Glu313 were involved for more than 20% of the time of the entire simulation period (Supplementary Figure S3B). Interestingly, Mortaparib^Mild^ was found to interact with the catalytic domain of PARP1 in a way similar to Olaparib (an established inhibitor of PARP1). It inhibited the catalytic site by forming firm interactions with residue Gly863 through a hydrogen bond, with residue Tyr896 through pi-pi interaction of aromatic ring, and with residues Lys903, Phe988, and Tyr 889 by hydrophobic interactions (Figure 2C). From the hydrogen bond occupancy plot of the simulation, it was found that residues Gly863, Tyr889, Tyr896, and Lys903 were making interactions for more than 25% of the simulation time (Supplementary Figure S3C). The interactions of Olaparib at Gly863, Lys903, and Tyr896 of PARP1 were observed at similar residues and found to be comparable with Mortaparib^Mild^. This confirmed that Mortaparib^Mild^ and Olaparib bind to the same catalytic region of PARP1 (Figure 2D).

The average structures from the simulation trajectories were used for MM/GBSA binding energy calculation. The binding energy of Mortaparib^Mild^ with the mortalin binding site of p53, the p53 binding site of mortalin, and the catalytic site of PARP1 was −55.43 kcal/mol, −55.96 kcal/mol, and −53.64 kcal/mol respectively. Based on this, it was concluded that Mortaparib^Mild^ possesses similar affinity towards the three tested targets (p53, mortalin, and PARP1).

### Mortaparib^Mild^-treated cells showed downregulation of mortalin and disruption of Mortalin-p53 complexes

**FIGURE 3:**
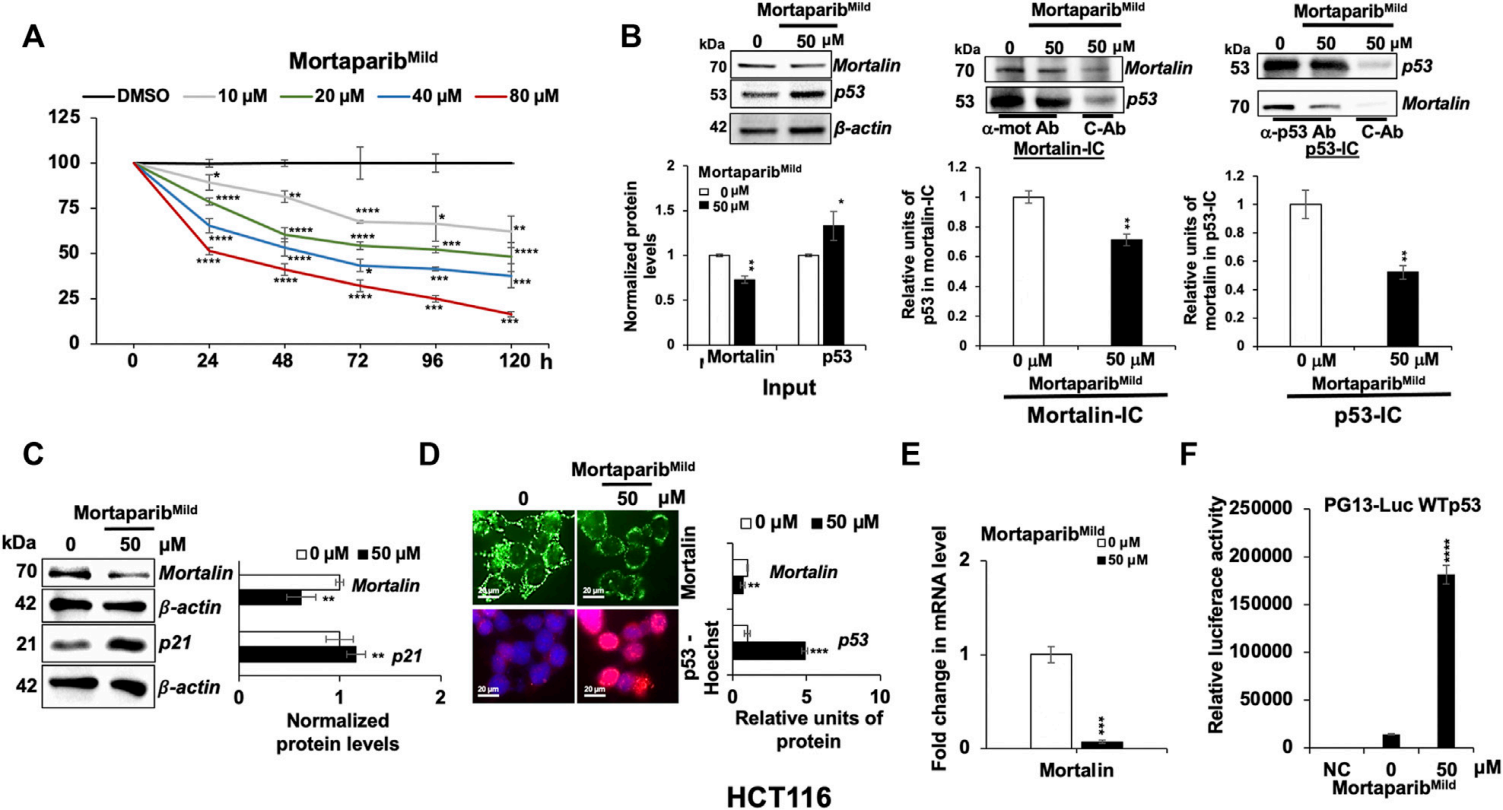
Mortaparib^Mild^ abrogated the interaction of mortalin and p53 in HCT116 (wild type p53) cells. **(A)** Mortaparib^Mild^ caused dose-dependent cytotoxicity in HCT116 cells. The MTT-based cell viability assay was performed after 24–120 h of treatment. **(B)** Mortaparib^Mild^ treated cells showed decrease in the level of mortalin and increase in the level of p53 (input lanes). Mortalin immunocomplexes (Mortalin-IC) showed a decrease in p53 in Mortaparib^Mild^-treated cells. p53-immunocomplexes (p53-IC) also showed decrease in mortalin in Mortaparib^Mild^-treated cells. **(C)** Western blot showing the expression level of mortalin and p21. Mortaparib^Mild^ treated cells showed decrease in mortalin (also seen in B) and increase in p21 (consequence of increase in p53). β-actin was used as an internal loading control. **(D)** Immunostaining of mortalin and p53 in control and treated cells showing decrease in mortalin and increase in nuclear p53 in treated cells. **(E)** mRNA expression of mortalin as determined by RT-qPCR showing decrease in mortalin and increase in p53 at mRNA level. **(F)** Wild type p53-driven luciferase reporter assay in negative control (NC, untransfected cells), control (transfected but untreated) and treated (transfected and treated with 50 μM Mortaparib^Mild^) cells. Data were normalized against control and plotted as fold difference. Each data set represented the mean ± SD of at least three independent experiments. Statistical significance was defined as values of *p* > 0.05 (ns), *p* ≤ 0.05 (*)‬, *p* ≤ 0.01 (**), *p* ‬≤ 0.001 (***), and *p* ≤ 0.0001 (****), which represent non-significant, significant‬, very significant, highly significant, and extremely significant, respectively.

We next examined if Mortaparib^Mild^ could function by independent mechanism(s) as well and therefore first determined the level of mortalin expression in control and Mortaparib^Mild^-treated cells. The doses were selected, based on independent cell viability assays that showed Mortaparib^Mild^ inhibited cell proliferation with IC_50_ of 50–80 μM for most cancer cell types (Supplementary Figure S4A). Based on the dose-dependent cytotoxicity assays, HCT116 (p53 wild type) and T47D (p53-mutant) cells were selected for the present study; cell viability assays for both cells (24–48 h) are shown in Supplementary Figure S4B,C. By multiple dose- and time-dependent cytotoxicity assays, we determined IC_50_, IC_30_, and IC_10_ concentrations for HCT116 and T47D cells. Of note, Mortaparib^Mild^ showed weaker cytotoxicity as compared to Mortaparib^Plus^ (Sari et al., 2021), and HCT116 cells were more responsive to Mortaparib^Mild^ than T47D cells (Supplementary Figure S4D,E). Based on these data, 50 μM (∼48 h; IC_50_) was selected for further analyses (Figure 3A).

First, to validate the *in-silico* results showing the ability of Mortaparib^Mild^ to interact with mortalin and p53, co-immunoprecipitation analyses were performed. As shown in Figure 3B, by Western blotting, decrease in mortalin and increase in p53 protein in Mortaparib^Mild^-treated cells was recorded. Mortalin-immunocomplexes probed with anti-p53 antibody revealed decrease in the amount of p53 in mortalin-complexes from Mortaparib^Mild^ treated HCT116 cells. In order to rule out the effect of unspecific immunoprecipitation of mortalin with IgG, we also obtained p53-immunocomplexes and probed them with anti-mortalin antibody. The data showed decrease in mortalin in p53-immunocomplexes in Mortaparib^Mild^ treated cells. Quantitation of the data from more than three experiments (Figure 3B) endorsed that Mortaparib^Mild^ caused disruption of mortalin-p53 interaction. To further support the ability of Mortaparib^Mild^ to disrupt mortalin-p53 interactions, and to exclude the effect of transcription, translation, post-translation as well as molecular signaling networks that operate in live cells, we treated cell lysates (prepared in NP-40 lysis buffer) with Mortaparib^Mild^. As shown in Supplementary Figure S5A–C, cell lysates treated with Mortaparib^Mild^ showed disruption of mortalin-p53 complexes as evidenced by decreased amount of mortalin in p53-immunocomplexes (Supplementary Figure S5A) as well as decrease amount of p53 in mortalin-immunocomplexes (Supplementary Figure S5B). Of note, the treated cell lysates showed higher amount of mortalin and p53 in supernatant after the precipitation of p53 and mortalin, respectively (Supplementary Figure S5C). These data reaffirmed that Mortaparib^Mild^ abrogated mortalin-p53 interaction. We also used T47D cells that harbor mutant p53 (Supplementary Figure S6A). Of note, Mortaparib^Mild−^ treated cells did not show increase in p53 level due to the stable nature and excessive accumulation of mutant p53 in these cells. However, decrease in mortalin (mRNA and protein; Supplementary Figure S6A,B) and mortalin-p53 complexes (Supplementary Figure S6A) was similar to HCT116 cells.

Next, further to the decrease in mortalin protein in Mortaparib^Mild^-treated cells as detected by Western blotting (Figure 3C), we performed immunostaining. As shown in Figure 3D, decrease in mortalin and increase in nuclear p53 in Mortaparib^Mild^-treated cells was clearly observed. Since the reduction in mortalin was anticipated to be independent of the binding of Mortaparib^Mild^ to either mortalin or p53, we performed RT-qPCR and found significant decrease in mortalin mRNA in Mortaparib^Mild^-treated cells (Figure 3E; Supplementary Figure S6B). We also examined the transcriptional activation function of p53 in control and Mortaparib^Mild^-treated cells by wild type p53-responsive luciferase reporter (PG13-luc) assay. As shown in Figure 3F, Mortaparib^Mild^-treated HCT116 (wild type p53) cells showed remarkable increase in luciferase reporter activity, indicating the transcriptional activation of p53, also supported by increase in the level of p21 protein (Figure 3C) and decrease in cell viability. Similar reporter assays performed in T47D (mutant p53) cells did not show any increase in reporter activity in Mortaparib^Mild^-treated cells (Supplementary Figure S6C). Of note, Mortaparib^Mild^ disrupted the p53 did not increase in T47D cells and caused relocation of mutant p53 in the nucleus (Supplementary Figure S6A,D). These data demonstrated that Mortaparib^Mild^-mediated reactivation of transcriptional activation of p53 was specific to wild type p53. However, T47D cells were responsive to Mortaparib^Mild^ and showed growth arrest in short- and long-term dose-dependent viability assays (Supplementary Figure S6E,F, respectively). Taken together, these data suggested that Mortaparib^Mild^ has capability to disrupt mortalin-p53 interaction and activate wild type p53 function. It may also work through mechanisms independent to its effects on mortalin-p53 binding and wild p53 activity.

### Mortaparib^Mild^ disrupted DNA damage repair signaling in cancer cells

**FIGURE 4:**
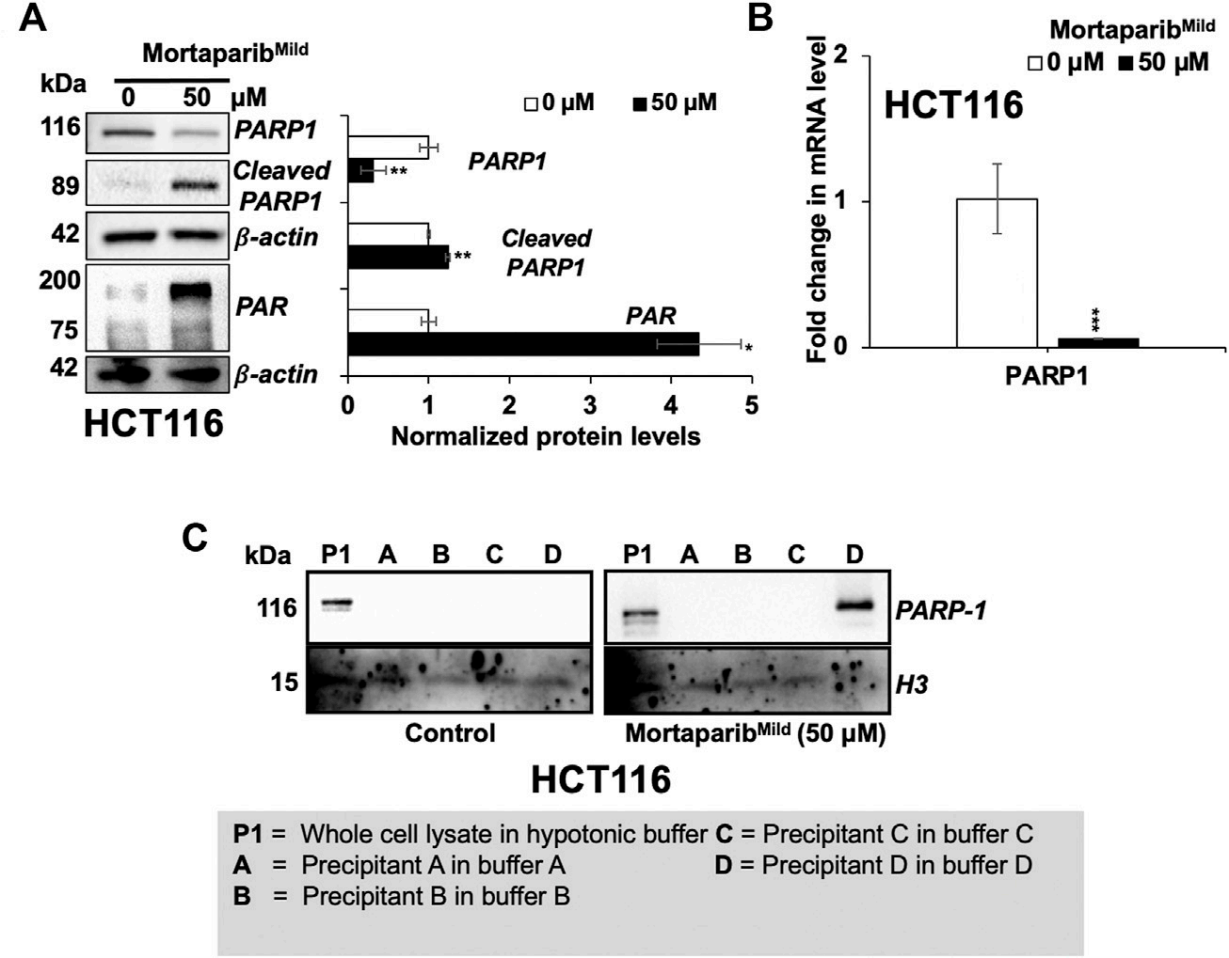
Mortaparib^Mild^ caused downregulation of PARP1 expression in HCT116 cells. **(A)** Western blot probed with anti-PARP1, anti-Cleaved-PARP1 and anti-PAR antibodies is shown. β-actin was used as an internal loading control. **(B)** mRNA expression of PARP1 as determined by RT-qPCR. **(C)** DNA trapping assay showing trapping of PARP1 in DNA in treated cells. Histone H3 was used as a loading control. Data were normalized against control and plotted as fold difference. Each data set represented the mean ± SD of at least three independent experiments. Statistical significance was defined as values of *p* > 0.05 (ns), *p* ≤ 0.05 (*)‬, *p* ≤ 0.01 (**), *p* ‬≤ 0.001 (***), and *p* ≤ 0.0001 (****), which represent non-significant, significant‬, very significant, highly significant, and extremely significant, respectively.

Based on our earlier findings on the effect of Mortaparib (Putri et al., 2019) and Mortaparib^Plus^ (Elwakeel et al., 2021; Sari et al., 2021) on PARP1 activity, we next investigated the expression levels of PARP1 and cleaved PARP1 in the Mortaparib^Mild^-treated HCT116 cells by Western blotting. Decrease in full-length PARP1 protein and mRNA was observed in Mortaparib^Mild^-treated cells (Figures 4A,B). Furthermore, the 89-kDa cleaved fragments of PARP1 and PAR were seen to be accumulated in Mortaparib^Mild^-treated cells (Figure 4A). Trapping assay in control and treated cells was performed to fractionate PARP1-DNA complexes in hypotonic buffers (A-D; Table 2) of variable stringency, wherein the tight complex is characterized by fractionation of PARP1 in higher stringency buffer as described earlier (Murai et al., 2012). The data showed fractionation of PARP1 from control cells in lower stringency (P1) and from treated cells in higher stringency buffer (D) suggesting that PARP1 was trapped into the DNA in the treated cells (Figure 4C) and hence may cause anomalous DNA repair. Although the direct experimental evidence to show the physical interaction of the compound with PARP1 and binding domain analyses remain to be clarified, these data demonstrated the potential of Mortaparib^Mild^ to impair DNA damage repair signaling in cancer cells.

### Mortaparib^Mild^ inhibits cancer cell proliferation, apoptosis and growth arrest

**FIGURE 5:**
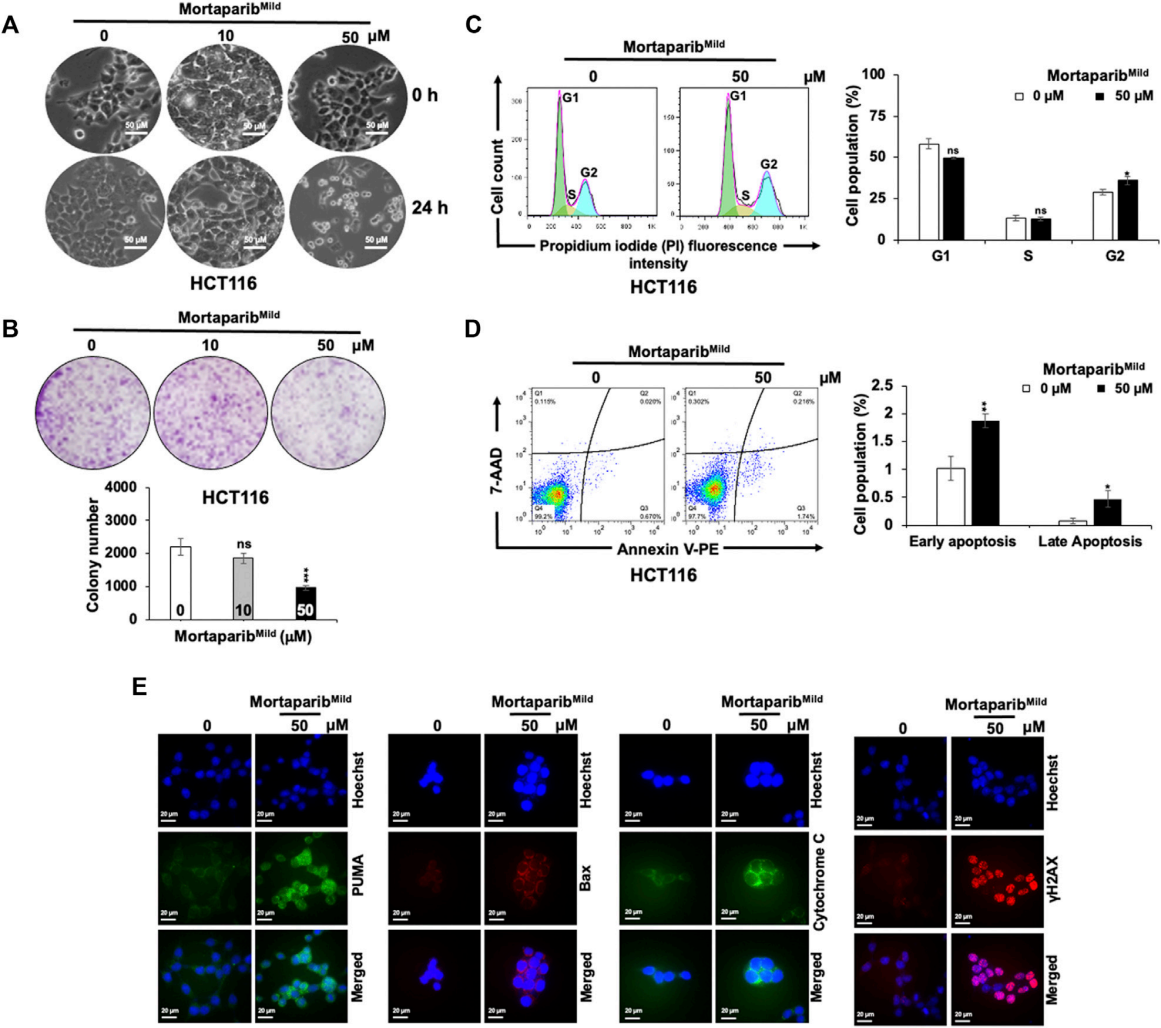
Mortaparib^Mild^ caused inhibition of cell proliferation in HCT116 cells. **(A)** Cell morphology of the control and treated (10 and 50 μM) cells showing growth arrest and apoptotic phenotypes post-24 h treatment is shown. **(B)** Colony forming efficiency in control and treated (10 and 50 μM; 10 days) showed dose-dependent decrease in colony forming efficiency. Mortaparib^Mild^ (50 μM) induced G2/M cell-cycle arrest **(C)** and apoptosis **(D)**. The latter was validated by increase in expression levels of protein involved in apoptosis (PUMA, Bax and Cytochrome C) and DNA damage accumulation (γH2AX). **(E)**. Each data set represented the mean ± SD of at least three independent experiments. Statistical significance was defined as values of *p* > 0.05 (ns), *p* ≤ 0.05 (*)‬, *p* ≤ 0.01 (**), *p* ‬≤ 0.001 (***), and *p* ≤ 0.0001 (****), which represent non-significant, significant‬, very significant, highly significant, and extremely significant, respectively.

Based on the above computational and experimental evidence, it was predicted that Mortaparib^Mild^ would inhibit cancer cells proliferation in p53-dependent and independent mechanisms. As described above, mutant p53 harboring T47D cells showed growth arrest (Supplementary Figure S6E,F). Consistently, as shown in Figure 5A, HCT116 cells treated with low (10 µM) and high (50 µM) concentration of Mortaparib^Mild^ for 24 h showed growth arrest and apoptotic phenotypes, respectively. In long-term clonogenic assays, HCT116 cells showed dose-dependent decrease in colony forming efficiency (Figure 5B). Whereas 10 μM Mortaparib^Mild^-treated (∼10 days) cells showed insignificant effect on colony forming efficiency, 50 μM Mortaparib^Mild^ caused about 50% reduction in the colony number. Cell cycle analysis in control and Mortaparib^Mild^-treated cells showed a dose-dependent increase in the percentage of cell population in G2 phase (Figure 5C), and apoptosis assay revealed increase in percentage of apoptotic cells in response to high dose (50 μM) of Mortaparib^Mild^ (Figure 5D). The effect was further confirmed by immunostaining of proteins involved in apoptosis (PUMA, Bax and Cytochrome C) (Figure 5E). Furthermore, consistent with the PARP-1 trapping data that suggested anomalous DNA repair in Mortaparib^Mild^-treated cells, γH2AX staining showed remarkable increase in the latter (Figure 5E).

### Mortaparib^Mild^ caused inhibition of cancer cell migration

**FIGURE 6:**
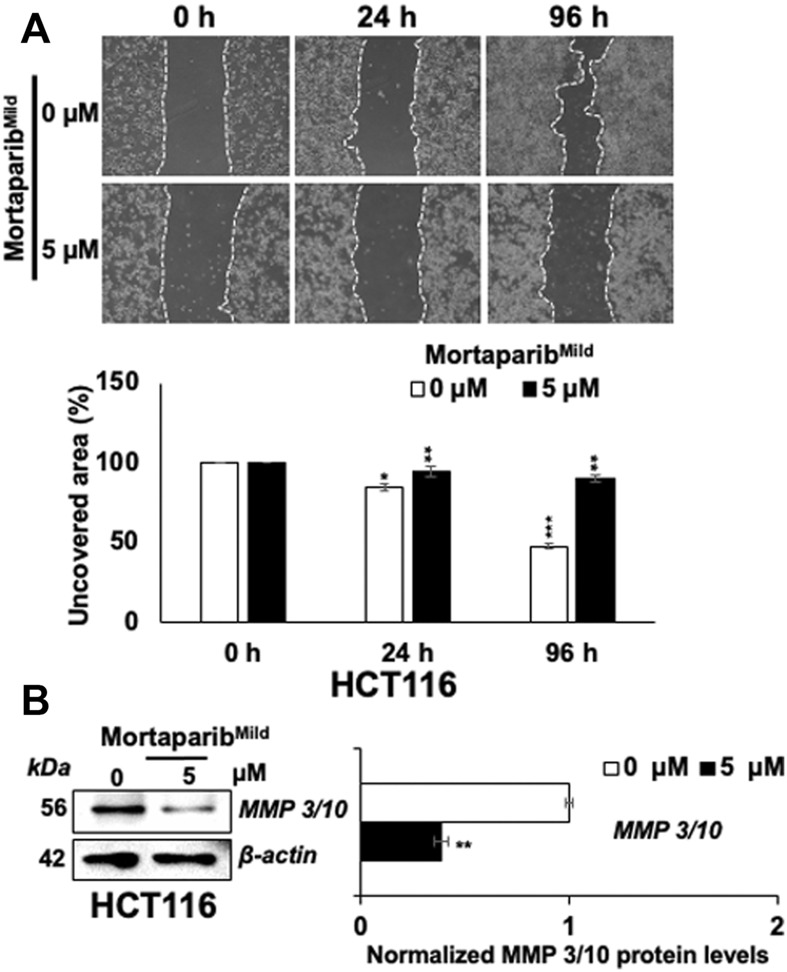
Mortaparib^Mild^ caused inhibition of cell migration in HCT116 cells. **(A)** Mortaparib^Mild^ (5 μM)-treated cells showed delay in migration in Scratch-wound assays. **(B)** Treated cells showed decrease in MMP 3/10 expression on Western blots wherein β-actin was used as an internal loading control. Each data set represented the mean ± SD of at least three independent experiments. Statistical significance was defined as values of *p* > 0.05 (ns), *p* ≤ 0.05 (*)‬, *p* ≤ 0.01 (**), *p* ‬≤ 0.001 (***), and *p* ≤ 0.0001 (****), which represent non-significant, significant‬, very significant, highly significant, and extremely significant, respectively.

Overexpression of mortalin and PARP1 has been shown to promote migration of cancer cells. Knock-down of these proteins has been connected to compromised migration ability of cancer cells (Schiewer and Knudsen, 2014; Na et al., 2016) by p53-independent mechanism. In light of this information and downregulation of mortalin and PARP1 in Mortaparib^Mild^-treated cells, we next examined the migration of cells by Scratch-wound assay in HCT116 as well as T47D cells. In order to rule out the effect on cell proliferation, extremely low non-toxic concentration of Mortaparib^Mild^ (5 μM) was used. As shown in Figure 6A and Supplementary Figure S7, Mortaparib^Mild^-treated both HCT116 and T47D cells showed delay in migration endorsing that the effect on cell migration involves p53-independent mechanism(s). Cancer cell migration is largely influenced by matrix metalloproteinases (MMPs) that are enriched in most cancers and function in the degradation of the extracellular matrix and non-matrix proteins (Jabłońska-Trypuć, et al., 2016). Earlier studies have shown upregulation of MMP3 in mortalin-enriched metastatic cancer cells (Wadhwa et al., 2006.) In light of this information, we examined the expression of MMP 3/10 in control and Mortaparib^Mild^-treated cells and found decrease in the latter (Figure 6B) suggesting it as one of the p53-independent mechanisms involved in Mortaparib^Mild^-mediated decrease in cancer cell migration.

## Discussion

Mortalin is a multifunctional stress chaperone that belongs to the family of heat shock (HSP70) proteins. It is enriched in cancer cells and inactivates tumor suppressor protein p53 by retaining it in the cell cytoplasm and thereby preventing its nuclear functions, essential for cell cycle arrest. Early on, mortalin was detected to be highly expressed in colorectal cancers and marked as an important target for drug therapy (Dundas et al., 2005). Abrogation of p53 and mortalin complex and reactivation of p53 function has been proposed as a viable strategy to identify drug candidates in mortalin-driven cancers. This strategy has been explored in drug screening for colorectal cancer (Sari et al., 2021). Several earlier studies have reported that the targeting of mortalin-p53 interaction by natural and chemical compounds causes nuclear translocation of p53 and reactivation of its transcriptional activation function leading to growth arrest in cancer cells (Wadhwa et al., 1998; Wadhwa et al., 2000; Wadhwa et al., 2002b; Kaul et al., 2005; Widodo et al., 2007a; Lu et al., 2011; Lu et al., 2011; Grover et al., 2012; Nigam et al., 2015). Two novel triazole derivatives, Mortaparib (Putri et al., 2019) and Mortaparib^Plus^ (Elwakeel et al., 2021; Sari et al., 2021) were earlier isolated by screening of a chemical library for compounds capable of disrupting mortalin-p53 interaction and causing nuclear translocation and activation of wild type p53 function. These compounds were shown to possess anticancer potential by *in vitro* and *in vivo* analyses that also demonstrated inactivation of PARP1 function. In the present study, we report the third novel triazole derivative that downregulated mortalin expression, disrupted mortalin-p53 complex yielding nuclear transport and activation of p53 and caused inactivation of PARP1 function. Based on these activities and milder cytotoxicity, the compound is named Mortaparib^Mild^.

By computational and molecular docking analyses, Mortaparib^Mild^ was predicted to bind to mortalin, and p53. Although the direct experimental evidence of the physical interactions of the compound with each of the target protein and binding domain analyses are yet to be clarified, co-immunoprecipitation of these proteins from control and treated cells, and cell lysates confirmed that Mortaparib^Mild^ was able to disrupt mortalin-p53 complexes both in live cells and cell lysates. To validate its p53-independent activity/multi-modal mechanism of action, its interaction with PARP1 was studied and compared with the known drug Olaparib. Our computational study highlighted that Mortaparib^Mild^ could inhibit PARP1 similar to Olaparib and suggested it to be a good drug candidate against colorectal cancer.

Expression analyses of mortalin and PARP1 on protein and mRNA level revealed that Mortaparib^Mild^ caused decrease in mortalin expression both at the protein and mRNA level (Figures 3C–E). This activity was independent to that of its binding to mortalin and p53 proteins. Furthermore, Mortaparib^Mild^ also caused downregulation of PARP1 expression, associated with increase in cleaved PARP1 and PAR (Figure 4A). The excessive activation of PARP1 and PAR accumulation leads to parthanatos, a unique cell death program (Wang et al., 2019). The accumulation of PAR polymer is considered as a hallmark for parthanatos or PARP1-mediated cell death (Fatokun et al., 2014). The trapping of PARP1 into the DNA in Mortaparib^Mild^-treated cells suggested its inhibitory effect on DNA repair yielding growth arrest. PARP1 inhibitors have been shown to sensitize cancer cells to other anticancer drugs and hence being used to overcome drug resistance of aggressive tumors (Murai et al., 2012; Liu et al., 2016). Of note, Mortaparib^Mild^ caused not only the growth arrest/apoptosis of cancer cells but also inhibited cell migration at a very low non-toxic concentration. Besides blocking the p53 function, overexpression of mortalin in cancers has been shown to contribute to malignancy and metastasis by its interactions with heterogenous ribonucleoprotein k (hnRNP-K), protein-kinases (MAP2K or MEK), and Raf/MEK/ERK (Wu et al., 2013; Ryu et al., 2014). Decrease in cell migration in Mortaparib^Mild^-treated cells was in line with these reports. Matrix metalloproteinases (MMPs) are responsible for multiple regulatory roles and enhance migration of cancer cells. MMPs expression (both at the protein and RNA levels) is commonly upregulated in cancer cells and hence suggested as cancer therapeutic targets (Gobin et al., 2019). MMP 3 and MMP 10 have been established as typical MMPs involved in metastatic properties of cancer cells (Tokito and Jougasaki, 2016). We found decrease in the expression of MMP 3/10 in Mortaparib^Mild^ -treated cells suggesting its ability to inhibit cancer cell migration, invasion, and metastasis. Taken together, we report a novel triazole, Mortaparib^Mild^ that targets mortalin and PARP1, causes activation of wild type p53 function in cancer cells and hence warrants further attention for elucidating its mechanisms of action (involving DNA damage, stress, tumor suppressor, metastatic and other signaling pathways), experimental validation of its anticancer activity in laboratory and clinic.

## Conclusion

Regulation of p53, mortalin, and PARP1 plays a significant role in normal cells by keeping cell proliferation in control. Such control is abolished in cancer cells wherein mortalin is overexpressed and inactivates p53. We discovered a novel compound, Mortaparib^Mild^, capable of disrupting mortalin-p53 interaction, as evidenced by extensive computational and experimental analyses. The p53-mortalin complex showed structural stability and was disrupted by Mortaparib^Mild^ that interacted with the binding domains of the two proteins. Furthermore, Mortaparib^Mild^ interacted with the catalytic site of PARP1 predicting its inactivation. Experimental analyses indeed endorsed activation of p53 function and inactivation of PARP1 yielding dose-dependent growth arrest/apoptosis in cancer cells. We propose Mortaparib^Mild^ as a new member of Mortaparib class of inhibitors that warrant further laboratory and clinic attention.

## Data Availability

The original contributions presented in the study are included in the article/Supplementary Material, further inquiries can be directed to the corresponding author.
