# Supplementary material for: Identification of a new member of Mortaparib class of inhibitors that target mortalin and PARP1
Source: Front Cell Dev Biol. 2022 Sep 12;10:918970. doi: 10.3389/fcell.2022.918970 (PMC9510692; doi:10.3389/fcell.2022.918970)

Supplementary File 1

Identification of a new member of Mortaparib class of inhibitors that target mortalin and PARP1

Hazna Noor Meidinna[1†], Seyad Shefrin[2†], Anissa Nofita Sari[1], Huayue Zhang[1], Jaspreet Kaur Dhanjal[3], Sunil C Kaul[1], Durai Sundar[2*] and Renu Wadhwa[1*]

[†]These authors have contributed equally to this work

[*]Correspondence: Durai Sundar (sundar@dbeb.iitd.ac.in) and Renu Wadhwa (renu-wadhwa@aist.go.jp).

Supplementary File 1 - Figure S1: (A) Interaction of Mortaparib^{Mild} with p53 in frames captured every 10 ns time interval till 100 ns. (B) Interaction of Mortaparib^{Mild} with mortalin in frames captured every 10 ns interval till 100 ns.

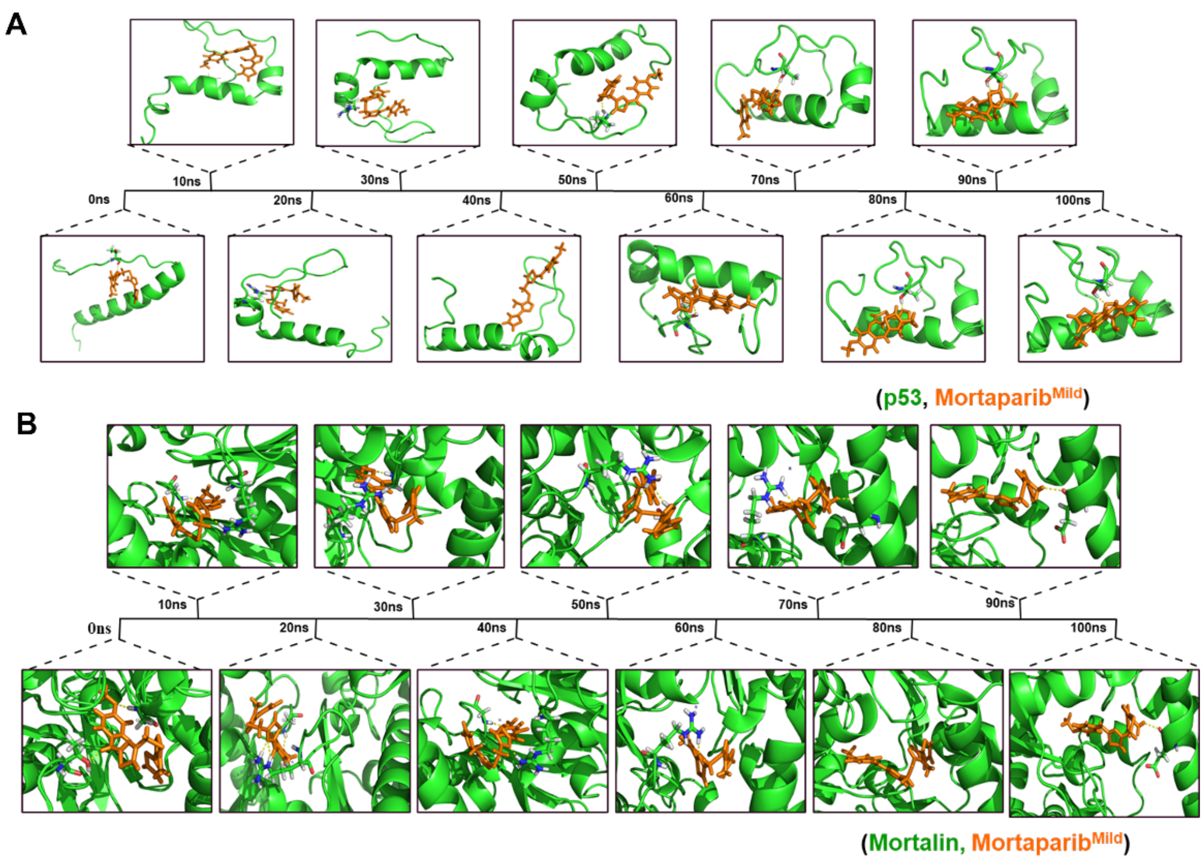

Supplementary File 1 - Figure S2: Interaction of Mortaparib^{Mild} with PARP1 in frames captured every 10 ns time interval till 100 ns.

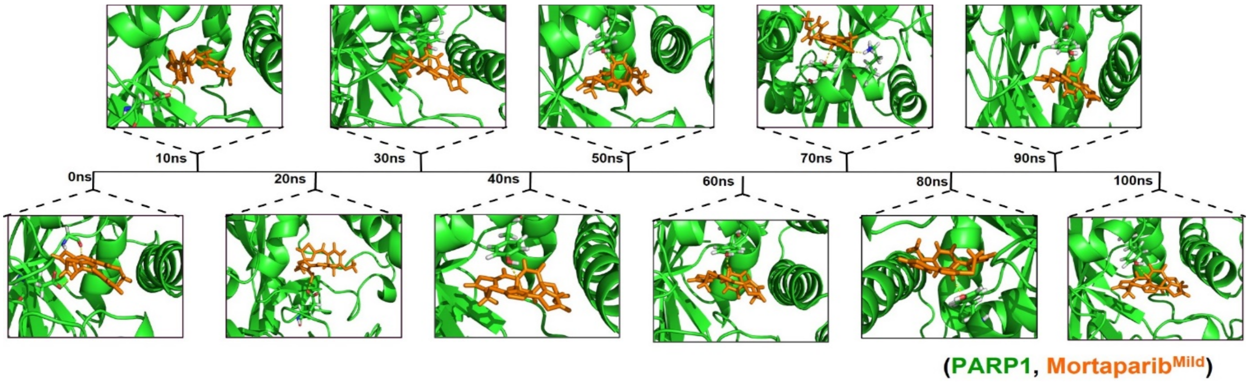

Supplementary File 1- Figure S3: Hydrogen bond occupancy of Mortaparib^{Mild} with target proteins throughout the simulation period (A) Mortaparib^{Mild}-p53 (B) Mortaparib^{Mild}-Mortalin (C) Mortaparib^{Mild}-PARP1 (Note: it is possible to have interactions with >100% as some residues may have multiple interactions of a single type with the same ligand atom).

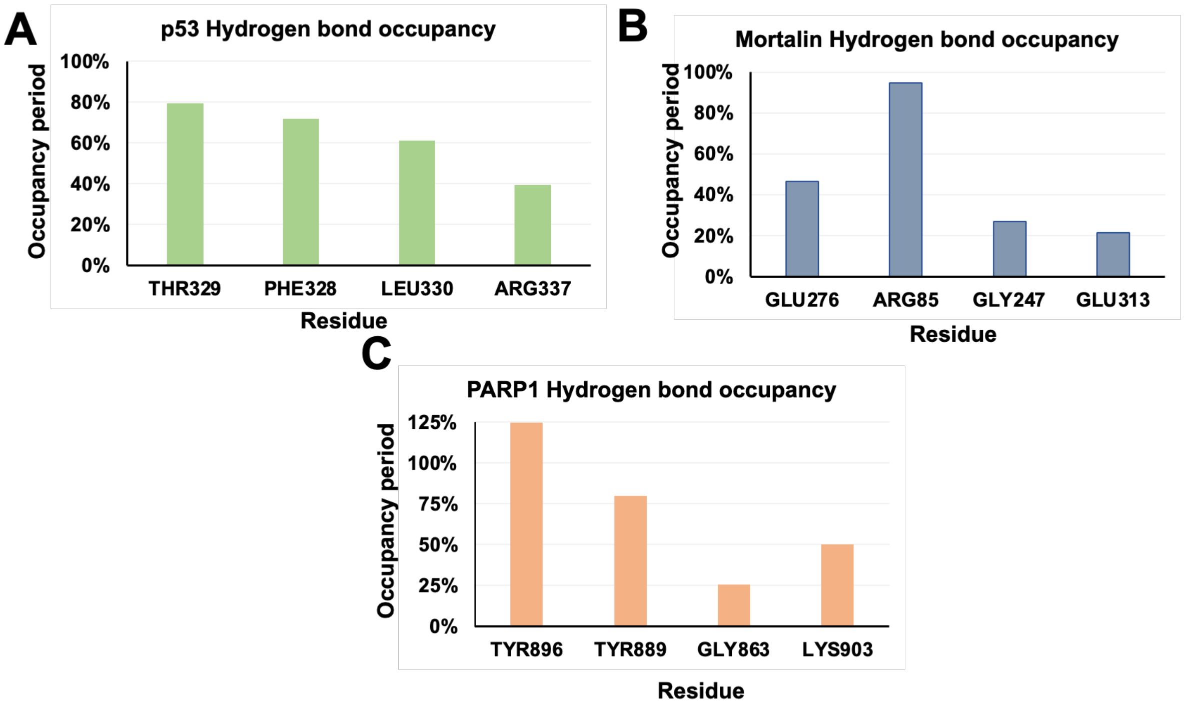

Supplementary File 1 - Figure S4: Mortaparib^{Mild} caused dose-dependent cytotoxicity to all the cancer cell lines tested, as shown (A). The MTT-based cell viability assay was performed in wild type p53 (HCT116) (B) and mutant p53 (T47D) (C) harboring cells after 24-48 h of treatment showed dose dependent cytotoxicity both at 24 and 48 h time points. Effect of Mortaparib^{Mild} was compared to that of Mortaparib^{Plus} wherein the latter showed stronger cytotoxicity in both cell lines (D and E). Data were normalized against control and plotted as a percentage difference. Each data set represented the mean ±SD of at least three independent experiments. Statistical significance was defined as values of $p > 0.05$ (ns), $p \leq 0.05$ (*), $p \leq 0.01$ (**), $p \leq 0.001$ (***), and $p \leq 0.0001$ (****), which represent non-significant, significant, very significant, highly significant, and extremely significant, respectively.

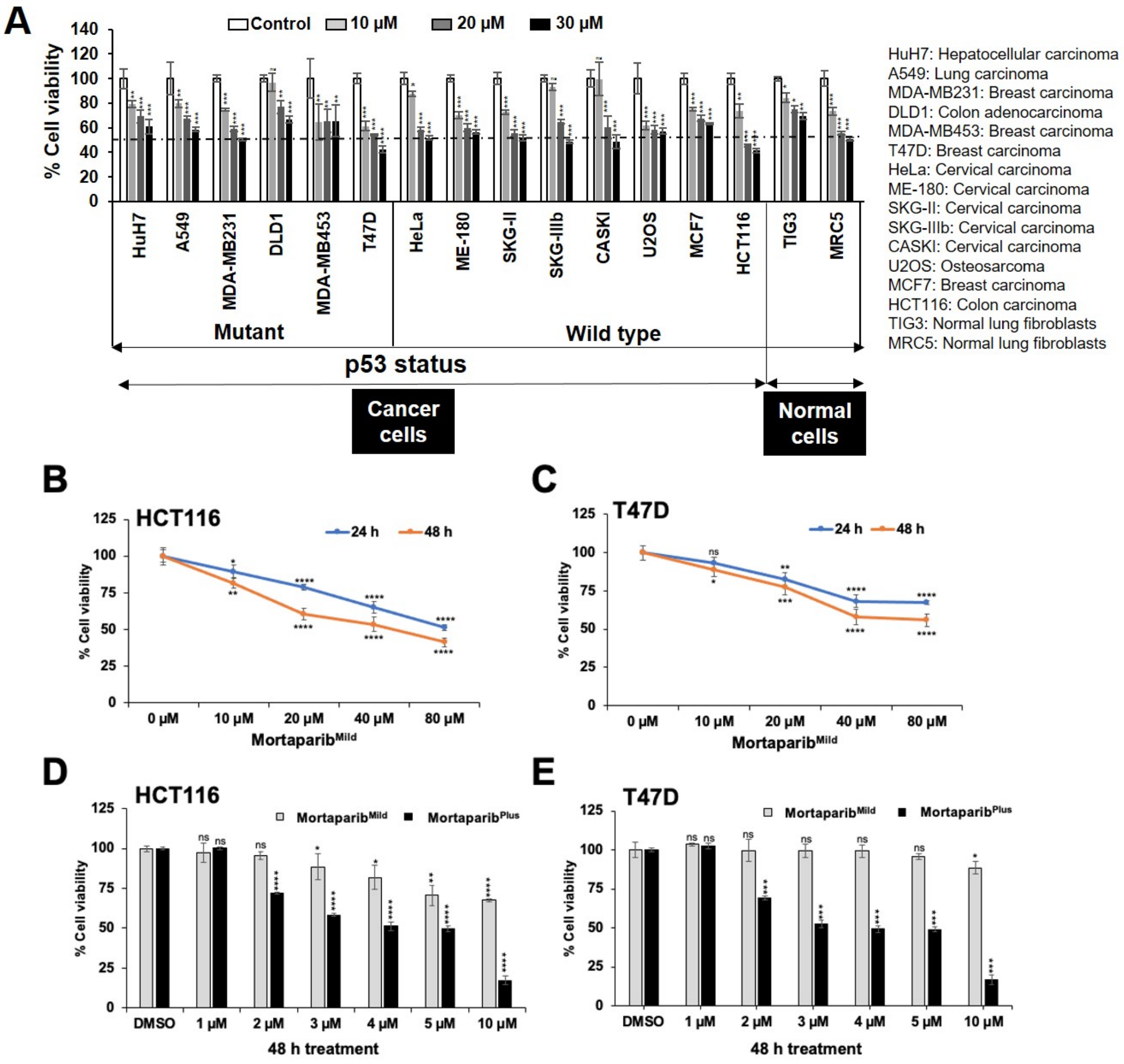

Supplementary File 1 - Figure S5: Mortaparib^{Mild} abrogated the interaction of mortalin and p53 in HCT116 cell lysates. HCT116 cell lysates were precleared with control IgG and then incubated with Mortaparib^{Mild} (DMSO was used as a solvent control) followed by immunoprecipitation with either mortalin or p53 and detection of coimmunoprecipitated p53 or mortalin, respectively, with specific antibodies as indicated in material and methods section. (A) p53-immunocomplexes (p53-IC) from Mortaparib^{Mild}-treated cell lysates showed decrease in mortalin. (B) Mortalin-immunocomplexes (Mortalin-IC) from Mortaparib^{Mild}-treated cell lysates showed decrease in p53. (C) The treated cell lysates showed higher amount of mortalin and p53 in supernatant after the precipitation of p53 and mortalin, respectively.

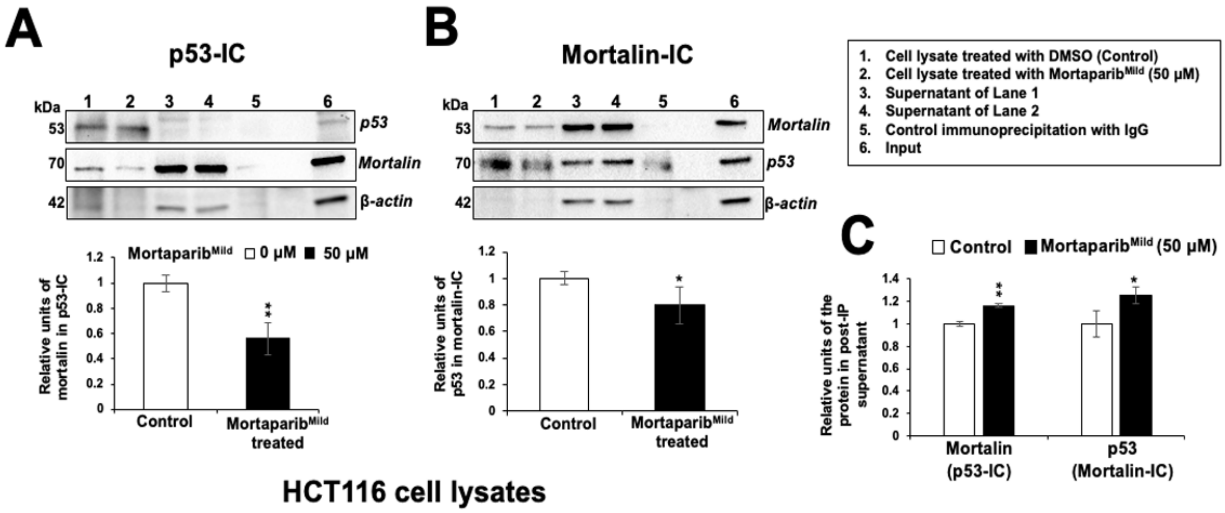

Supplementary File 1 - Figure S6: Mortaparib^{Mild} abrogated the interaction of mortalin and p53 in T47D cells. (A) Mortalin immunocomplexes (Mortalin-IC) showed a decrease in p53 in Mortaparib^{Mild}-treated cells. Decrease in mortalin level and an increase in p53 level in the input lanes from the same cell lysates was observed. (B) RT-qPCR showed decrease in mortalin mRNA in Mortaparib^{Mild}-treated cells. (C) Wild type p53-driven luciferase reporter assay in control and treated cells did not show any increase in the latter. Negative control (NC, untransfected cells), control (transfected but untreated - 0 µM) and treated (transfected and treated with 10 µM and 50 µM Mortaparib^{Mild}) cells are shown. Nuclear enrichment of mutant p53 was observed in treated cells (D). (E) Mortaparib^{Mild} caused inhibition of cell proliferation. Cell morphology of the control and treated (10 µM and 50 µM) cells showed growth arrest and apoptotic phenotypes, respectively. (F) Colony forming efficiency in control and treated (10 µM and 50 µM; 10 days) cells showed dose-dependent decrease. Each data set represented mean ± SD in at least three independent experiments. Statistical significance was defined as values of $p > 0.05$ (ns), $p \leq 0.05$ (*), $p \leq 0.01$ (**), $p \leq 0.001$ (***), and $p \leq 0.0001$ (****), which represent non-significant, significant, very significant, highly significant, and extremely significant, respectively.

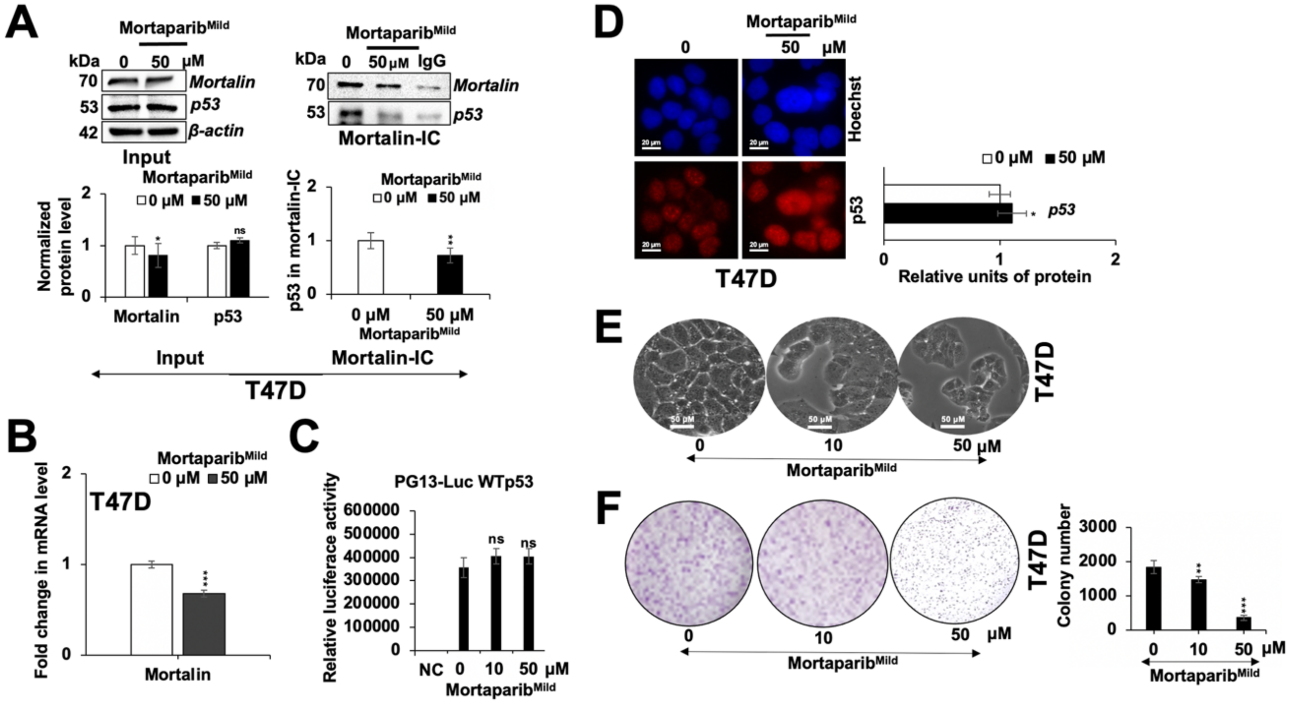

Supplementary File 1 - Figure S7: Mortaparib^{Mild} caused inhibition of cell migration in T47D cells. Mortaparib^{Mild} -treated (5 μM) cells showed delay in migration in Scratch-wound assays. Each data set represented the mean ± SD in at least three independent experiments. Statistical significance was defined as values of $p > 0.05$ (ns), $p \leq 0.05$ (*), $p \leq 0.01$ (**), $p \leq 0.001$ (***), and $p \leq 0.0001$ (****), which represent non-significant, significant, very significant, highly significant, and extremely significant, respectively.

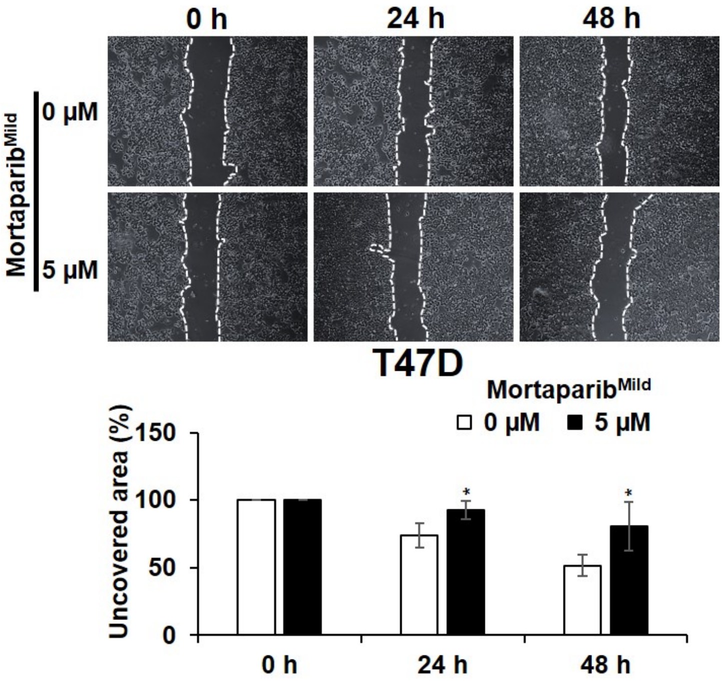

Supplement: Supplementary file 1 [file DataSheet1.pdf]
